# Advances in immunotherapeutic strategies for colorectal cancer commentary on: tumoral immune cell exploitation in colorectal cancer metastases can be targeted effectively by anti-CCR5 therapy in cancer patients by Halama et al

**DOI:** 10.1186/s40425-016-0197-y

**Published:** 2016-12-20

**Authors:** Dustin A. Deming

**Affiliations:** 1 Division of Hematology and Oncology, Department of Medicine, University of Wisconsin-Madison, Madison, WI USA; 2 University of Wisconsin Carbone Cancer Center, Madison, WI USA; 3 McArdle Laboratory for Cancer Research, Department of Oncology, University of Wisconsin-Madison, Madison, WI USA; 4 William S Middleton Memorial Veterans Hospital, Madison, WI USA

## Abstract

Colorectal cancer is a leading cause of cancer-related death in the United States, despite recent advances in treatment strategies. The immune system has been implicated in the pathogenesis of colorectal cancer, with numerous studies identifying either antagonistic or pro-tumorigenic effects of infiltrating immune cells. Therapeutic strategies harnessing the immune system to target cancers have evolved expediently over the last 5 years, especially the use of checkpoint inhibitors. Recently, a subset of patients whose colorectal cancers harbor a deficiency in mismatch repair proteins have demonstrated dramatic and durable response to checkpoint blockade. Unfortunately, the vast majority of colorectal cancers are mismatch repair proficient and resistant to these inhibitors. The tumor microenvironment has been implicated in the resistance to checkpoint block and ways to overcome these resistance mechanisms would be a major advance for the treatment of colorectal cancer. Here we provide commentary on a manuscript from Halama et al. examining CCL5/CCR5 as an immune biomarker and the potential role of anti-CCR5 agents for the treatment of patients with colorectal cancer.

## Background

Over the last 5 years, the use of immunotherapeutics has gained significant enthusiasm for the treatment of many cancers. In colorectal cancer (CRC), the immune system plays an important role, as multiple studies have demonstrated the prognostic importance of immune cell infiltration [1–4]. The immunoscore—which categorizes the location, type, and quantity of tumor-infiltrating lymphocytes (TILs)—has been revealed as one of the strongest prognostic markers in CRC. The immunoscore quantifies the density of cytotoxic (CD8^+^) and memory (CD45RO^+^) T cells (CD3/CD45RO, CD3^+^/CD8^+^ or CD8^+^/CD45RO^+^) located in the center and invasive margins of CRC cancers [1, 5]. Higher immunoscores, indicating increased infiltration of CD8^+^ and CD45RO^+^ T lymphocytes into the invasive margin and central tumor, are strong positive prognostic markers [1].

Agents targeting PD-1/PD-L1 or CTLA4 have demonstrated robust benefit for multiple cancer types, but their efficacy was limited in CRC until tested in the setting of high microsatellite instability (MSI-H). Pembrolizumab, an anti-PD-1 checkpoint inhibitor, resulted in an exciting objective response rate and overall survival (OS) in a subset of CRC patients with mismatch repair deficiency (dMMR) [6]. In an updated analysis presented at the American Society of Clinical Oncology annual meeting, patients with dMMR CRC had a response rate of 50% and a disease control rate of 89% compared to no response observed in the mismatch repair proficient cancers [7]. Impressively, patients with dMMR treated with pembrolizumab have durable responses with 61% without progression after 24 months. These tumors are characterized by deficiency in mismatch repair proteins resulting in high mutational burdens and the potential for increased neoantigen presentation. However, a high mutational load alone does not determine responses to checkpoint immunotherapy. In addition, since MSI-high is seen in only ~4% of metastatic CRC, further investigations are required to better understand how immunotherapies can be used for MSS CRCs.

Interestingly, MSI status and quantification of the number of TILs do not fully predict outcomes in patients with CRC. A cohort of CRCs has been described that contain a signature characterized by high lymphoid gene expression, and somewhat unexpectedly, was associated with a worse prognosis [8]. These CRCS also have an increased mesenchymal and myeloid infiltration. A mesenchymal signature was also recently reported in a cohort of melanoma patients who were considered resistant to checkpoint blockade [9]. This signature consists of genes associated with the epithelial-to-mesenchymal transition, immunosuppressive genes, and monocyte/macrophage chemotactic genes.

While manipulation of specific subsets of immune cells can lead to tumor response, as above, Halama and colleagues have identified an immunologically distinct microenvironment at the invasive margins of CRC liver metastases in the setting of increased macrophage-related cytokines and chemokines [10]. Increased levels of CCL5, which is secreted by T cells, were identified at the invasive margin. The production of CCL5 results in tumor cell proliferation, invasion and increased production of matrix metalloproteinases by tumor-associated associated macrophages. These investigators then sought to determine if maraviroc, a CCR5 inhibitor originally developed for the treatment of HIV, would have anti-neoplastic properties through inhibition of CCL5/CCR5 signaling using CRC tumor explants. Morphologic changes consistent with tumor cell death were observed in addition to mitigation of tumor-promoting cytokines. Further investigation as part of a phase I clinic trial was performed in a total of 14 patients with late-line treatment-refractory metastatic CRC. Pre- and post-treatment biopsies were obtained for pharmacodynamic analyses and demonstrated that maraviroc as a single agent reduced tumor proliferation, reduced certain cytokines and caused morphologic changes consistent with tumor cell death. This therapy was well-tolerated. Clinically, however, as a single agent no responses were observed and the median PFS was only 1.15 months for the core cohort and 1.55 months for the extension cohort. Interestingly though, 5 patients went on to receive maraviroc in combination with chemotherapy, presumably chemotherapy they had previously been resistant to, and 3 out of these 5 patients had objective partial responses.

Improved treatment strategies for patients with CRC are clearly needed and immunotherapeutic approaches are highly promising. Unfortunately, MSS CRCs have been largely resistant to immunotherapeutic approaches, such as single agent checkpoint blockade. The tumor microenvironment has been implicated in this resistance in multiple studies including this study by Halama and colleagues. This study, however, has multiple caveats. This is a very small study that unfortunately did not meet its primary endpoint of demonstrating efficacy with CCR5 inhibition. The most intriguing story in this manuscript relates to the potential of CCR5 inhibition to result in re-sensitization to chemotherapy. The exact mechanism by which this might occur is unknown though presumably occurs as a result of mitigation of the tumor promoting effects of CCR5 signaling from the microenvironment surrounding the cancer. This could include alterations in signaling through chemokines, cytokines and growth factors. The potential of CCR5 to sensitize CRCs to chemotherapy would be further supported had the authors confirmed that the patients in the expanded cohort had previously progressed on the chemotherapy used. It is also important to know how long it had been since the patients were exposed to these regimens. Tumors that progressed on a regimen less than 6 months ago would not be expected to respond, though if it had been longer than that a low yet significant number of those cancers can respond to retreatment.

## Conclusion

Interesting activity has been demonstrated with CCR5 inhbiition in CRC. These results deserve further evaluation in larger, better controlled, and more homogeneous subsets of patients with metastatic treatment refractory colorectal cancer.

## Abbreviations

CRC: Colorectal cancer
MSI: Microsatellite instability
MSS: Microsatellite stable
ORR: Objective response rate
OS: Overall survival
PFS: Progression-free survival
TIL: Tumor-infiltrating lymphocyte
